# Jiedu Granule Combined with Transcatheter Arterial Chemoembolization and Gamma Knife Radiosurgery in Treating Hepatocellular Carcinoma with Portal Vein Tumor Thrombus

**DOI:** 10.1155/2019/4696843

**Published:** 2019-06-25

**Authors:** Jianchu Wang, Jinhong Luo, Xiaolan Yin, Wei Huang, Huangming Cao, Guilin Wang, Jincheng Wang, Jun Zhou

**Affiliations:** ^1^Department of Hepatobiliary Surgery, Affiliated Hospital of Youjiang Medical University for Nationalities, Baise, China; ^2^Department of Oncology, Shanghai East Hospital, Tongji University School of Medicine, Shanghai, China; ^3^Department of Radiotherapy, Changhai Hospital Hongkou District Affiliated to Naval Medical University, Shanghai 200081, China; ^4^Liver Transplantation Center, The First Affiliated Hospital of Nanjing Medical University, Nanjing, China

## Abstract

**Background:**

The potential advantages of Jiedu granule (a compound Chinese herbal medicine) combined therapeutic strategies compared with non-Jiedu granule therapeutic strategies for hepatocellular carcinoma (HCC) with portal vein tumor thrombosis (PVTT) remain unclear. Thus, the purpose of the study was to investigate the safety and efficacy of Jiedu granule for HCC with PVTT.

**Methods:**

We retrospectively reviewed the clinical data of 190 patients (94 for non-Jiedu and 96 for Jiedu) with HCC and PVTT from March 2012 to October 2016. Patients were followed up by outpatient examination and telephone till November 2018.

**Results:**

It was statistically insignificant between the two groups in baseline characteristics. Procedure-related adverse events (AEs) were observed and compared and most of them were not serious which were easily controlled or subsided naturally. No AE-induced death happened. The median overall survival (OS) rates in the single TACE plus GKR and Jiedu granule combined group were 11.3 months (95% CI: 9.168-13.435) and 15.8 months (95% CI: 13.244-18.339), respectively (*p* = 0.00047).

**Conclusions:**

Jiedu granule combined with TACE plus GKR is safe in HCC patients with PVTT and this Chinese herbal medicine is worthy to be promoted because of better prognosis which needs further research.

## 1. Introduction

Approximately 75-85% liver cancers were hepatocellular carcinoma (HCC), and it was reported as the sixth most frequently diagnosed tumor (841000 new cases) and the fourth major cause of cancer death (782000 deaths) worldwide in 2018 [1]. It was reported in 2015 that there were 466.1 thousand estimated new cases and 422.1 deaths in China [2]. HCC has a peculiarity to invade the hepatic vasculature macroscopically or microscopically, which definitely contributes to the unfavorable prognosis for HCC [3–5]. Portal vein tumor thrombosis (PVTT) is recognized as the main form of macrovascular invasion of HCC. Numerous studies [6–8] have shown that 10-60% of HCC patients are diagnosed with PVTT simultaneously. According to the Barcelona Clinic Liver Cancer Staging (BCLC) [9], HCC with PVTT is classified as stage C and the median survival is just 2.7 months without any interventions [10, 11].

Sorafenib was recommended as the only standard treatment for HCC-PVTT patients [12]. However, several comparative studies [13, 14] have shown that sorafenib can only prolong nearly 3-month lifetime of patients with advanced HCC. In addition, it is impossible for many patients to afford the sorafenib because of its high price especially in China. Therefore, it is necessary to find alternative and relatively inexpensive therapeutic options for HCC patients with PVTT.

Our previous studies [15, 16] have shown that gamma knife radiosurgery (GKR) is safe and effective for HCC patients with PVTT and combination therapy (i.e., transarterial chemoembolization (TACE) plus GKR) can significantly prolong patients' life compared with TACE alone. A recent study [17] also reported that TACE plus radiotherapy can provide a 1.6-month survival benefit compared with sorafenib in treating HCC with macroscopic vascular invasion. Therefore, it should be noticed that TACE plus GKR became control treatments in this study, although it was a nonstandard control. Jiedu granule is a kind of traditional Chinese medicine that has been reported to show positive effects combined with TACE on unresectable hepatocellular carcinoma [18–21]. This separate study aimed to investigate the safety and efficacy of Jiedu granule on HCC-PVTT patients who received combined therapy (i.e., TACE plus GKR).

## 2. Materials and Methods

### 2.1. Study Design and Patients Enrollment

This retrospective study was approved by the Institutional Ethics Committee of Changhai Hospital (Shanghai, China). Liver biopsy was used to determine the diagnosis of HCC according to the European Association for the Study of the Liver (EASL) recommendations [12]. Contrast-enhanced computed tomography (CT) or magnetic resonance imaging (MRI) was used to confirm PVTT due to the appearance of intraluminal filling defect and typical arterial enhancement [22, 23].

We retrospectively reviewed 376 HCC patients with PVTT who were admitted to Changhai Hospital (Shanghai, China) between March 29^th^, 2012, and October 17^th^, 2016. The inclusion criteria were applied in the selection of patients: (1) age 18 to 75; (2) no therapeutic interventions before hospitalizing in the Changhai Hospital; (3) ECOG (Eastern Cooperative Oncology Group) performance status (PST): 0-2; (4) Child-Pugh score 5-7; (5) adequate hematologic and renal functions; (6) received combination treatments (TACE plus GKR) alone or plus Jiedu granule administration. The exclusion criteria were as follows: (1) received other treatments such as sorafenib, TACE or GKR alone, hepatic resection, and radiofrequency ablation; (2) extrahepatic metastases; (3) cirrhosis with signs such as obstinate ascites and hepatic encephalopathy; (4) received multiple episodes of TACE or GKR; (5) inadequate clinical data.

#### 2.1.1. Treatment Procedures

Treatment strategies were determined according to patients' clinical pathological characteristics, which were evaluated by the Changhai Hospital HCC Expert Team (composed of hepatologists, radiation oncologists, interventional radiologists, and Traditional Chinese Medicine (TCM) physicians). All patients signed informed consents before they received treatments.

Traditional combination treatments comprised TACE and GKR in the control group. TACE was performed bimonthly or trimonthly according to specific conditions of patients. Subsequent GKR was performed 7-10 days after the last TACE.

TACE was performed by delivering an emulsion of lipiodol (10-20 ml) and pirarubicin (30 mg, produced by Good Manufacturing Practice certificated Wanle Pharmaceutical Factory, Shenzhen, China; Production License No.1106C6, 1304C6, and 1501C4) into the selected vessels (proper or right/left hepatic artery decided by radiography) feeding the tumor followed by embolization using absorbable particles (gel foam).

GKR was performed using Treatment Planning System (TPS, OUR New Medical Technologies Co. Ltd., Shenzhen, China) by two radiation oncologists who delineated the irradiation area of each patient by contrast-enhanced CT scan or MRI. The gross tumor volume (GTV) including tumor thrombosis and the primary tumor in the liver was delineated using above-mentioned image techniques. If it was a long distance between the primary lesion and tumor thrombosis or if multiple lesions existed in the liver, they were then included into different target regions. We used TPS to define a 5-10 mm margin around the GTV as the planning target volume (PTV). The median tumor margin dose was 40 Gy (ranging from 35 to 45 Gy), with a median isodose line of 55% (ranging from 50% to 60%). Because of different adjacent normal tissue tolerances, the dose prescription was limited and the liver and adjacent normal tissues were delineated during the target planning process. Moreover, at least 1/3 of the liver volume should be spared. Dose-volume histograms were harnessed to protect adjacent normal tissues. GKR was performed using a stereotactic body radiotherapy system called Gamma Master Space Body Knife System (also named OUR-QGD system, OUR New Medical Technologies Co. Ltd., Shenzhen, China). The full course of gamma knife radiotherapy was performed in 12-16 days (once every other day).

The Jiedu granule added in the observation group (combined with TACE plus GKR) consisted of the traditional Chinese herbs shown as follows: root of* Salvia chinensis*, root of* Actinidia valvata*, gizzard membrane of* Gallus gallus domesticus*, and bulb of* Cremastra appendiculata* (1:1:0.4:0.4). In the Jiedu group, 5.9 g Jiedu granule (equivalent to 78.6 g raw herbal material, produced by Tianjiang Pharmaceutical Factory, Jiangsu, China; Production License No.1212042, 1307045, 1403107, 150436, and 1603068) was administered with hot water twice a day, half an hour after meals. Patients in the Jiedu group started oral administration of Jiedu granule after a week of TACE and insist on it for about 42 days (a course of treatment).

#### 2.1.2. Patient Follow-Up and Data Collection

Posttreatment follow-ups were performed through telephone interview and outpatient review 1 month after leaving hospital and every 3 months subsequently, using physical examinations, CT, or MRI and serum biochemical analysis. Treatment-related adverse events (AEs) were observed and recorded until 3 months after GKR based on National Cancer Institute Common Terminology Criteria for Adverse Events (CTCAE) v5.0 [24]. It should be noticed that survival time was reckoned from the day of initial hospitalization to the day of death.

#### 2.1.3. Statistical Analyses

The Student t-test or permutation test was applied for continuous data. The Wilcoxon test was for ranked data and Chi-Square test or Fisher exact test was applied for categorical data. Median overall survival (mOS) was calculated using the Kaplan-Meier method and compared using the log-rank test. In Cox proportional hazards model, variables with* P* values < 0.2 on univariate analyses were selected in multivariate analysis.* P* < 0.05 was considered statistically significant. All statistical analyses were conducted using R 3.5.1.

## 3. Results

### 3.1. Baseline Characteristics

**Figure 1:**
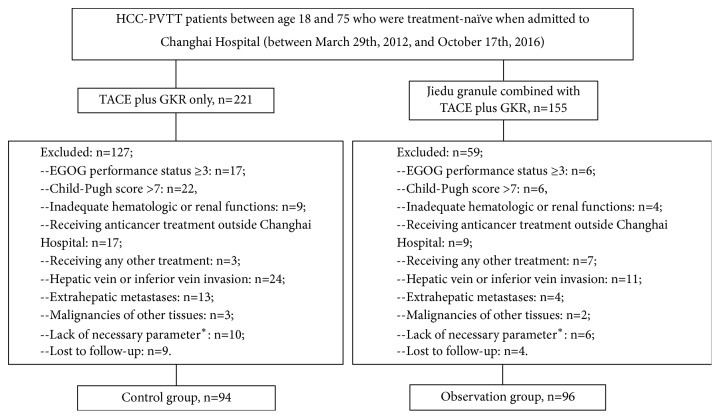
*Flowchart of patient inclusion and exclusion*. *∗*: referring to patients whose clinical/laboratory follow-ups were incomplete that impeded subsequent analyses.

In the beginning, there were 376 hospitalized HCC patients with PVTT in the Changhai Hospital (Shanghai, China). 190 patients were finally included into this study (Figure 1), with 94 in the combined therapy (TACE plus GKR) group and 96 in the Jiedu granule plus combined therapy group. It was statistically insignificant in baseline characteristics between these two groups (Supplement Material 1).

### 3.2. Safety and Procedure-Related Adverse Events

No significant direct adverse effects associated with Jiedu granule were reported in the Jiedu group. 4 (4.2%) patients reported mild diarrhea after administration of Jiedu granule. This symptom was improved after the beginning of symptomatic treatments such as bland diet and electrolyte replacement in the observation group.

Common AEs related to TACE-GKR in both groups were summarized in Tables 1 and 2. Most of them were not serious (grade 1-2) and remitted spontaneously or were controlled after symptomatic treatments. There were no significant differences in terms of procedure-related adverse events (Tables 1 and 2) between these two groups. Compared with pre-procedure, it was statistically significant in both groups that cases of liver function impairment in 3 months post-procedure became more (Table 2). No post-procedure deaths (within 4 weeks) occurred in both groups.

### 3.3. Survival Analyses

**Figure 2:**
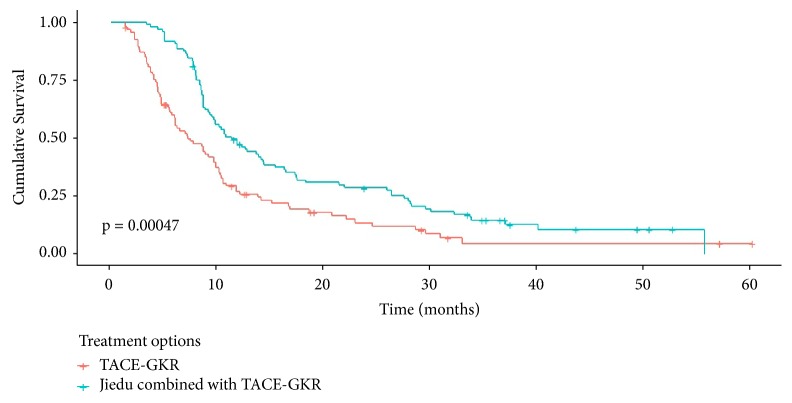
*Kaplan-Meier survival curves of patients by treatment options*. GKR: gamma knife radiosurgery; TACE: transarterial chemoembolization.

27 patients (14 in the Jiedu group and 13 in the TACE-GKR group) kept alive at the latest follow-up (November 4^th^ 2018). As is shown in Figure 2 and Supplement Material 2, the median overall survival (OS) rates of patients in the TACE-GKR and Jiedu-TACE-GKR group were 11.3 months (95% CI: 9.168-13.435) and 15.8 months (95% CI: 13.244-18.339), respectively (*p *= 0.00047). Jiedu combined therapy produced significant survival benefits (hazards ratio [HR], 0.540; 95%CI: 0.396-0.739;* p* < 0.001) compared with TACE-GKR alone on multivariate analysis (Supplement Material 2). In addition, Child-pugh A (HR: 0.724; 95%CI: 0.590-0.878;* p* < 0.001), PST 0-1 (HR: 0.595; 95%CI: 0.367-0.896;* p* < 0.001), and tumor diameter ≤ 5 cm (HR: 0.595; 95%CI: 0.367-0.896;* p* < 0.001) were significant advantageous indicators of overall survival versus Child B, PST 2, and tumor diameter >10 cm, respectively (Supplement Material 2).

Subgroup (according to PVTT type) multivariate analyses revealed that Jiedu granule produced survival benefits both in patients with branch of PVTT (HR: 0.644; 95% CI: 0.445-0.932;* p *= 0.02) and in patients with main PVTT (HR: 0.496; 95% CI: 0.263-0.932;* p *= 0.03).

Multifactors statistical analysis adopted in the Jiedu granule group revealed that Child-pugh A (HR: 0.413; 95% CI: 0.323-0.785;* p *= 0.016), PST 0-1 (HR: 0.479; 95% CI: 0.279-0.826;* p *= 0.008), and AFP ≤ 400 (HR: 0.408; 95% CI: 0.206-0.785;* p *= 0.019) were significant advantageous indicators of overall survival versus Child-pugh B, PST 2, and AFP > 400, respectively.

## 4. Discussion

The treatment strategies specially for HCC-PVTT patients have always become as a difficult and unsolved problem in clinical practices. A general consensus has been reached in academic circles that combination therapy has great potential to derive novel therapeutic strategies on HCC with PVTT in the future [25, 26]. This retrospective study investigated the safety and efficacy of Jiedu granule combined with TACE plus GKR on HCC with PVTT.

Although many patients receiving either of combination treatments suffered procedure-related AEs which have been summarized in Tables 1 and 2, most of these AEs were not serious and got remission with symptomatic treatments easily. Few patients suffered severe post-procedure AEs and no death occurred in both groups. Results of AEs indicate that TACE plus GKR can be safe whether or not combined with Jiedu granule in treating HCC-PVTT patients.

Survival analyses were conducted to reveal that Jiedu granule combined treatments provided a 4.5-month survival benefit compared with TACE-GKR alone (Figure 2, Supplement Material 2). Moreover, Jiedu granule produced survival benefits whether patients were with branch of PVTT or with main PVTT. In addition to treatment option, other factors including Child-pugh score, ECOG PST, and tumor size (maximum diameters) impacted overall survival in our study, which is analogous to the results of our previous research or other similar studies [15, 27, 28].

TACE plus GKR is a nonstandard therapy in this study, but we have verified its curative effect in our previous studies [15, 16]. We further developed a tri-combination therapy in which Jiedu granule was added based on TACE plus GKR. This study initially confirmed the safety and efficacy of the novel combination therapy. We also realize some limitations in our study. First, the concrete mechanism of Jiedu granule improving prognosis of HCC-PVTT patients remains unclear which needed further basic research such as in vitro studies (proliferation, apoptosis effects) for the intricate components of Jiedu granule. Second, this study with small sample size limited the persuasion of the results. The consistent results with bootstrap and permutation test which are suitable for small samples were calculated and we planned to collect more data for this study in the future. Third, the major defect of its study is that it is a retrospective study which limited the robustness of related data. Therefore, we should conduct more comprehensive and detailed prospective studies with large sample sizes to verify the tri-combination therapy we proposed. In addition, the TACE plus Jiedu granule or GKR plus Jiedu granule should also be tested and compared with the tri-combination treatment strategy in terms of curative effects and safety in order to develop the optimum treatment strategy (minimum treatment with better outcome).

In conclusion, this study primarily verified the tolerability and efficacy of Jiedu granule combined with TACE plus GKR in treating HCC with PVTT. Our results indicate that Jiedu granule combined with TACE plus GKR is safe and can prolong survival in HCC-PVTT patients. Future studies are needed to corroborate the positive effects of Chinese herbs in combination therapy for HCC-PVTT patients and determine whether inexpensive Jiedu granule can be recommended as a conventional treatment for HCC patients with PVTT.

## Figures and Tables

**Table 1:** Main procedure-related clinical adverse events by CTCAE grades in both groups.

CTCAE	Group	Grade 1	Grade 2	Grade 3	Grade 4	Grade 5	All grades	*P* value
Abdominal pain	TACE-GKR (n=94)	10(10.64)	1(1.06)	0	0	0	11(11.7)	0.430
	Jiedu plus TACE-GKR (n=96)	8(8.33)	0	0	0	0	8(8.33)	
Anorexia	TACE-GKR (n=94)	12(12.77)	14(14.89)	4(4.26)	0	0	30(31.91)	0.301
	Jiedu plus TACE-GKR (n=96)	8(8.33)	15(15.63)	1(1.04)	0	0	24(25)	
Ascites	TACE-GKR (n=94)	3(3.19)	1(1.06)	0	0	0	4(4.26)	0.991
	Jiedu plus TACE-GKR (n=96)	2(2.08)	2(2.08)	0	0	0	4(4.17)	
Constipation	TACE-GKR (n=94)	3(3.19)	1(1.06)	0	0	0	4(4.26)	0.967
	Jiedu plus TACE-GKR (n=96)	4(4.17)	0	0	0	0	4(4.17)	
Dermatitis	TACE-GKR (n=94)	12(12.77)	4(4.26)	0	0	0	16(17.02)	0.728
	Jiedu plus TACE-GKR (n=96)	14(14.58)	1(1.04)	0	0	0	15(15.63)	
Fatigue	TACE-GKR (n=94)	14(14.89)	15(15.96)	0	0	0	29(30.85)	0.362
	Jiedu plus TACE-GKR (n=96)	16(16.67)	9(9.38)	0	0	0	25(26.04)	
Fever	TACE-GKR (n=94)	21(22.34)	4(4.26)	0	0	0	25(26.6)	0.755
	Jiedu plus TACE-GKR (n=96)	17(17.71)	6(6.25)	0	0	0	23(23.96)	
Ileus	TACE-GKR (n=94)	6(6.38)	0	0	0	0	6(6.38)	0.144
	Jiedu plus TACE-GKR (n=96)	2(2.08)	0	0	0	0	2(2.08)	
Nausea/vomiting	TACE-GKR (n=94)	24(25.53)	5(5.32)	0	0	0	29(30.85)	0.143
	Jiedu plus TACE-GKR (n=96)	19(19.79)	1(1.04)	1(1.04)	0	0	21(21.88)	
Pneumonitis	TACE-GKR (n=94)	1(1.06)	3(3.19)	0	0	0	4(4.26)	0.401
	Jiedu plus TACE-GKR (n=96)	0	2(2.08)	0	0	0	2(2.08)	

CTCAE: National Cancer Institute Common Terminology Criteria for Adverse Events. GKR: gamma knife radiosurgery. TACE: transarterial chemoembolization.

**Table 2:** Main procedure-related laboratory adverse events by CTCAE grades in both groups.

CTCAE	Group	Pre-procedure				3 months post-procedure				*P* value*∗*	*P* value#
		All grades	*P *value	Grade≥3	*P *value	All grades	*P *value	Grade≥3	*P *value		
Leukocytopenia	TACE-GKR (n=94)	12(12.77)	0.448	0	1	16(17.02)	0.373	3(3.19)	0.721	0.539	0.246
	Jiedu plus TACE-GKR (n=96)	8(8.33)		0		11(11.46)		5(5.21)		0.628	0.059
HB	TACE-GKR (n=94)	11(11.70)	0.775	0	1	9(9.58)	0.964	1(1.06)	0.495	0.813	0.301
	Jiedu plus TACE-GKR (n=96)	9(9.38)		0		8(8.33)		0(0.00)		0.998	1
Thrombocytopenia	TACE-GKR (n=94)	10(10.64)	0.998	0	1	19(20.21)	0.943	3(3.19)	0.998	0.106	0.246
	Jiedu plus TACE-GKR (n=96)	10(10.42)		0		18(18.75)		4(4.17)		0.152	0.121
Hypoalbumin	TACE-GKR (n=94)	12(12.77)	0.780	0	1	17(18.09)	0.978	0(0.00)	1	0.419	1
	Jiedu plus TACE-GKR (n=96)	10(10.42)		0		14(14.58)		0(0.00)		0.513	1
ALT/AST↑	TACE-GKR (n=94)	37(39.36)	0.680	0	1	54(57.45)	0.551	14(14.89)	0.742	0.019	0.009
	Jiedu plus TACE-GKR (n=96)	34(35.42)		0		50(52.08)		17(17.71)		0.029	<0.001
TB↑	TACE-GKR (n=94)	23(24.47)	0.806	0	1	40(42.55)	0.757	4(4.26)	0.719	0.013	0.121
	Jiedu plus TACE-GKR (n=96)	26(27.08)		0		44(45.83)		3(3.13)		0.011	0.246
GGT↑	TACE-GKR (n=94)	49(52.13)	0.671	0	1	55(58.51)	0.790	18(19.15)	0.529	0.463	<0.001
	Jiedu plus TACE-GKR (n=96)	54(56.25)		0		59(61.46)		23(23.96)		0.558	<0.001
Creatinine	TACE-GKR (n=94)	0(0.00)	0.497	0	1	3(3.19)	0.759	1(1.06)	0.495	0.246	0.998
	Jiedu plus TACE-GKR (n=96)	2(2.08)		0		4(4.17)		0(0.00)		0.683	1
INR	TACE-GKR (n=94)	7(7.45)	0.998	0	1	13(13.83)	0.940	0(0.00)	0.497	0.237	1
	Jiedu plus TACE-GKR (n=96)	8(8.33)		0		14(14.58)		2(2.08)		0.257	0.497

*∗*: intragroup differences in all grades laboratory adverse events between baseline and month 3 were analysed with Exact McNemar's test; p<0.05 was considered statistically significant. #: intragroup differences in grade≥ laboratory adverse events between baseline and month 3 were also analysed with Exact McNemar's test. TB: total bilirubin; ALT: alanine transaminase; AST: aspartate transaminase; INR: international normalized ratio; GGT: gamma-glutamyl transpeptidase; CTCAE: National Cancer Institute Common Terminology Criteria for Adverse Events; GKR: gamma knife radiosurgery; TACE: transarterial chemoembolization.

## Data Availability

The original data is not available because of patient privacy.

## Supplementary Materials

Supplementary Materials Supplement Material 1: it enumerated the baseline characteristics of patients included in this study before receiving treatments to ensure the comparability of these two groups patients. Supplement Material 2: it described univariate and multivariate survival analyses by baseline characteristics and treatment options to mainly indicate that the second treatment option had better prognosis.
